# Mean platelet volume and red blood cell distribution width is associated with prognosis in premature neonates with sepsis

**DOI:** 10.1515/med-2021-0323

**Published:** 2021-08-25

**Authors:** Na Cai, Zhi Qiang Chen, Min Tao, Wen Ting Fan, Wei Liao

**Affiliations:** Department of Pediatrics, The First Hospital Affiliated to Army Medical University, Chongqing 400038, China

**Keywords:** premature neonates, sepsis, MPV, RDV, prognosis

## Abstract

**Objective:**

To evaluate the prognostic value of the mean platelet volume (MPV) and red blood cell distribution width (RDW) in sepsis among premature neonates.

**Methods:**

This was a retrospective cohort study conducted in the neonatal intensive care unit between May 2015 and May 2020. Premature neonates with late-onset sepsis were enrolled. The demographic data, blood cell count analysis, C-reactive protein, and blood culture were compared between survivors and non-survivors.

**Results:**

A total of 73 premature neonates with sepsis in the survivor group and 10 cases in the non-survivor group. Significant differences were observed between the survivor and non-survivor groups with regard to birth weight, MPV, and RDW (*P* < 0.05). The results of binomial stepwise logistic regression suggested that MPV (OR = 3.226, *P* = 0.017 < 0.05) and RDW (OR = 2.058, *P* = 0.019 < 0.05) were independent predictor for prognosis in preterm with sepsis. A receiver operating characteristic analysis showed that the areas under the curves were 0.738 for MPV alone, 0.768 for RDW alone, and 0.854 for MPV combined with RDW.

**Conclusion:**

MPV and RDW were independent predictors of prognosis and the combination of the two helps in predicting the prognosis of preterm with late-onset sepsis in the early stage.

## 1 Introduction

Sepsis is one of the most common infectious diseases of newborns, which mostly occurs in premature babies and low birth weight. Sepsis is the third leading cause of neonatal deaths with high morbidity and mortality, especially in developing countries [1,2], accounting for more than 25% of neonatal deaths [3]. The most important approach is to make an early assessment of the prognosis of sepsis and intervene as soon as possible to reduce the mortality. Currently, procalcitonin (PCT), interleukin-6 (IL-6), heparin-binding protein (HBP), C-reactive protein (CRP), serum resistin, and other indicators can be used to assess the severity and prognosis of sepsis [4,5,6,7,8]. However, these indicators have not been implemented in grassroots hospitals in many developing countries, and these tests also take a long time to get results, which may delay the diagnosis and treatment of children. Therefore, our research focuses on searching for effective indicators that are applicable to primary hospitals in developing countries and can quickly obtain results.

The mean platelet volume (MPV) and red blood cell distribution width (RDW) as a part of the complete blood count analysis are commonly measured, inexpensive, and widely available. Many studies showed that MPV and RDW were related to inflammatory diseases. MPV is an immediate indicator of platelet activation that is driven by inflammatory processes. Some studies in adults have revealed the relationship between MPV and prognosis in infectious diseases, including sepsis [9,10,11]. RDW is a parameter that measures the range of variation of red blood cell size. Elevated RDW was associated with outcome in pediatric critical illness [12]. Moreover, a few studies showed that RDW was associated with the mortality of sepsis in adults [13,14]. Until now, MPV and RDW are widely used to evaluate the prognosis in many diseases of adults and children. But, the effect on the prognosis of late-onset sepsis in premature neonates remains unclear. This study investigated the value and significance of MPV combined with RDW to assess the prognosis among premature neonates with late-onset sepsis, which may provide a new idea for evaluating the prognosis of late-onset sepsis.

## 2 Materials and methods

### 2.1 Study design

The study was conducted in the neonatal intensive care unit (NICU). Premature neonates with late-onset sepsis (age >3 days) diagnosed by a neonatal specialist at the First Hospital Affiliated to Army Medical University, China, between May 2015 and May 2020 according to expert consensus on diagnosis and treatment of neonatal sepsis (2019) [15] were enrolled in this study. Diagnostic criteria for sepsis are shown in Table 1. Depending on whether death occurred, premature neonates were divided into the survivor group and non-survivor group. Exclusion criteria included the following: (1) intrauterine infection leads to early-onset sepsis (age ≤3 days); (2) those with genetic metabolic diseases (neonates will be screened for genetic metabolism 3 days after birth) and congenital deformity; (3) infusion of red blood cells and platelets; (4) use of drugs that can cause changes in red blood cell morphology (such as erythropoietin, folic acid, vitamin B12, etc,); (5) blood system diseases; and (6) incomplete case data.

**Table 1:** Expert consensus on the diagnosis and management of neonatal sepsis (version 2019)

Confirm diagnosis	Clinical diagnosis
Blood culture was positive	Blood non-specific test was positive (at least 2 items)
Culture of the aseptic body cavity was positive	Cerebrospinal fluid test showed purulent meningitis
	DNA of pathogenic bacteria was detected in blood

Diagnostic criteria for sepsis: neonates have fever or low body temperature, cry less, poor mental response, and other clinical manifestations. At the same time, have at least the above one. Blood non-specific test includes ① blood cell count analysis: the white blood cell (WBC) count <5 × 10^9^/L, or WBC >20 × 10^9^/L; ② cell classification: immature neutrophils/total neutrophils (I/T) ≥0.16; ③ platelet (PLT) count <100 × 10^9^/L; ④ C-reactive protein (CRP) ≥8 mg/L.

This study was approved by the Ethics Committee of the First Hospital Affiliated to Army Medical University. All methods were performed in accordance with the relevant guidelines and regulations.

### 2.2 Data collection

Demographic data for all the study patients were obtained from the electronic medical records and included gender, gestational age, birth weight, age of onset, blood cell count analysis, blood culture, etc. When patients had clinical manifestations of sepsis (such as fever, poor feeding, and mental response), the blood samples collected for the first time would be examined. Laboratory parameters such as white blood cell (WBC) count, hemoglobin concentration (HC), platelet count (PLT), MPV, RDW, CRP, and blood culture were measured.

### 2.3 Statistical analysis

The categorical variables were compared using the Chi-square test or Fisher’s Exact test, and the continuous variables were compared using *T*-test or Mann–Whitney *U*-tests. Logistic regression analysis was used to assess independent predictors for death. The receiver operating characteristic (ROC) method was conducted to evaluate the utility of different variables in predicting prognosis in premature neonates with sepsis. ROC areas under the curve (AUCs) and cut-off points based on maximizing the sum of sensitivity and specificity were calculated. SPSS 20.0 (Inc., Chicago, IL, USA) was used to perform the statistical analyses with *P* < 0.05 considered to be statistically significant.

## 3 Results

### 3.1 Baseline characteristics

A total of 83 patients were included in the study. We analysed the baseline data of children with sepsis, including their gender, gestational age, birth weight, age of onset, small for gestational age infants (SGA), parenteral nutrition and mechanical ventilation. No significant differences were observed regarding these data among the survivor group and the non-survivor group (*P* > 0.05), except for birth weight (*P* < 0.05, Table 2).

**Table 2:** Baseline clinical characteristics of the survivor and the non-survivor groups

	Survivor (*n* = 73)	Non-survivor (*n* = 10)	*P*
Gender (male, *n*%)	39 (53.42)	5 (50.00)	1.000
Gestational age (week)	32.00 ± 1.71	31.17 ± 1.51	0.129
Birth weight (g)	1560.00 (1365.00–1925.00)	1380.00 (1032.50–1477.50)	0.013
Age of onset (days)	19.00 (12.50–24.50)	12.50 (9.00–26.00)	0.378
SGA (*n*%)	**3 (30.00)**	**18 (24.70)**	**0.708**
Parenteral nutrition (*n*%)	**10 (100.00)**	**57 (78.10)**	**0.197**
Mechanical ventilation (*n*%)	**3 (30.00)**	**7 (9.6)**	**0.097**

SGA, small for gestational age infants. Continuous variables of normal distribution were presented as mean ± SD, continuous variables of non-normal distribution were presented as medians (quartile ranges), and *T-*test or Mann–Whitney *U*-tests were applied to determine the group difference. Categorical variables were presented as absolute number and percentage and were compared using the *T-*test and Chi-square test, respectively. *P* < 0.05 was considered significant.

### 3.2 Significant differences in MPV and RDW

Significant differences were observed in MPV and RDW between the survivor and non-survivor groups (Table 3). MPV and RDW were significantly lower in the former than in the latter. No significant difference in WBC, PLT, HC, CRP, and positive rate of blood culture was observed among the two groups.

**Table 3:** Comparison of complete blood count analysis and blood culture between the two groups

	Survivor (*n* = 73)	Non-survivor (*n* = 10)	*P*
WBC (×10^9^/L)	9.90 (4.59–15.96)	8.95 (7.05–13.54)	0.933
PLT (×10^9^/L)	167.00 (107.50–267.50)	176.5 (113.00–252.75)	0.933
HC (g/L)	118.00 (105.00–129.50)	111.00 (106.25–117.50)	0.144
MPV (fL)	10.70 (10.20–11.15)	11.40 (10.53–12.25)	0.015
RDW (%)	15.40 (14.95–16.15)	16.55 (15.65–18.83)	0.006
CRP (mg/L)	14.03 (5.00–29.01)	19.30 (5.50–54.41)	0.483
Blood culture (positive, *n* %)	29 (39.73)	3 (30.00)	0.734

WBC, white blood cell; PLT, platelets; HC, hemoglobin concentration; MPV, mean platelet volume; RDW, red blood cell distribution width; CRP, C-reactive protein. Continuous variables were presented as medians (quartile ranges). The non-parametric Mann–Whitney *U-*test was applied to determine the group difference. Categorical variables were presented as absolute numbers and percentages, and were compared using Fisher’s Exact test. *P* < 0.05 was considered significant.

### 3.3 MPV and RDW were independent predictors for prognosis

Binomial stepwise logistic regression was used with whether death occurred as a dependent variable, and the above statistically significant indicators, including birth weight, MPV, and RDW were used as independent variables to test whether each factor had a significant effect on death. Hosmer–Lemeshow test showed that the regression model fits well (*P* = 0.174 > 0.05). The results suggested that MPV (OR = 3.226, *P* = 0.017 < 0.05) and RDW (OR = 2.058, *P* = 0.019 < 0.05) were independent predictor for prognosis in preterm neonates with sepsis. Birth weight was not an independent predictor for prognosis (OR = 0.998, *P* = 0.167 > 0.05). High MPV and RDW were independent risk factors for death in preterm neonates with sepsis (Table 4).

**Table 4:** Logistic regression analysis results

Variables	*b*	SE (*b*)	Walds	*P*	OR	95% CI for OR
MPV	1.171	0.489	5.738	0.017	3.226	(1.237–8.411)
RDW	0.721	0.308	5.486	0.019	2.058	(1.125–3.757)
Birth weight	**–0.002**	**0.002**	**1.908**	**0.167**	**0.998**	**(0.994–1.001)**

MPV, mean platelet volume; RDW, red blood cell distribution width.

### 3.4 The high predictive value of MPV combined with RDW for the prognosis of neonates with sepsis

**Figure 1:**
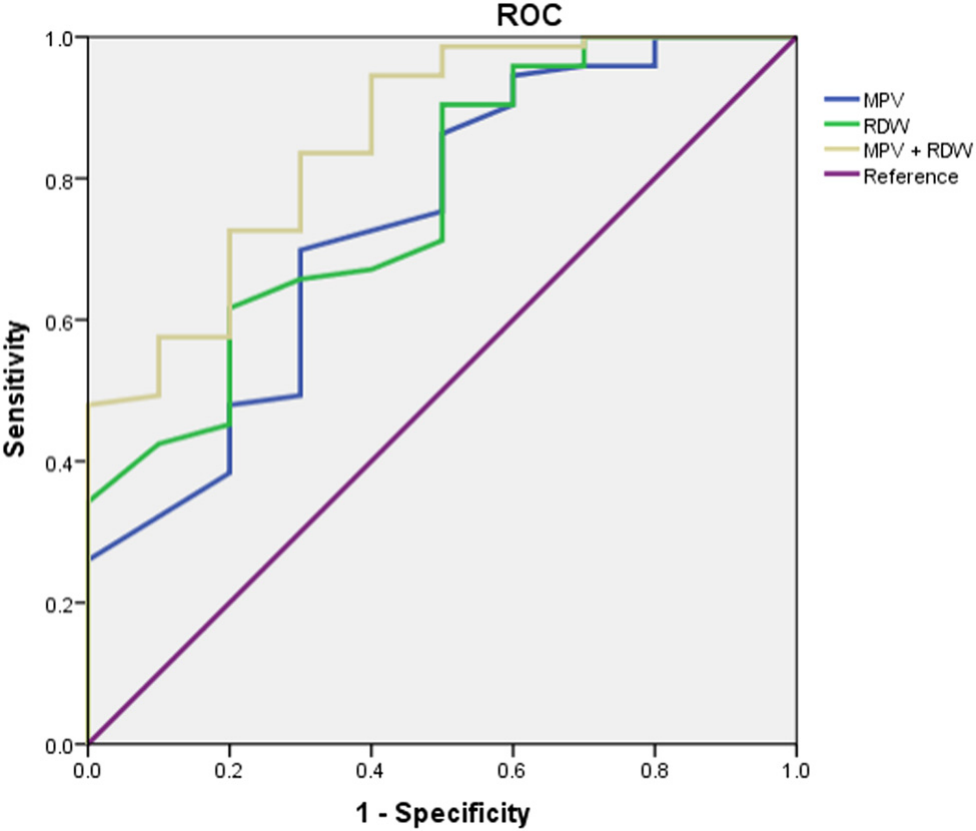
The ROC curve of MPV combined with RDW for the prognosis of children with sepsis.

95% CI was 0.568–0.907 for MPV alone, 0.617–0.919 for RDW alone, and 0.732–0.976 for MPV combined with RDW (Table 5). The ROC analysis of sepsis showed that AUC was 0.738 for MPV alone, 0.768 for RDW alone, and 0.854 for MPV combined with RDW, suggesting that MPV combined with RDW is better for predicting prognosis in sepsis neonates (Table 5 and Figure 1).

**Table 5:** The AUC of MPV combined with RDW

	AUC	*P*	Cut-off value	Sensitivity	Specificity	Youden index	95% CI
MPV (fL)	0.738	0.015	10.95	0.70	0.70	0.40	**0.568–0.907**
RDW (%)	0.768	0.006	15.75	0.80	0.62	0.42	**0.617–0.919**
MPV (fL) + RDW (%)	0.854	0.000	11.70, 15.80	0.84	0.70	0.54	**0.732–0.976**

MPV, mean platelet volume; RDW, red blood cell distribution width.

## 4 Discussion

Neonatal sepsis remains a major cause of mortality and morbidity in the NICU worldwide. It is important to identify the determinants that predict the severity and prognosis of neonatal sepsis, so as to deal with such patients in a timely manner.

In recent years, PCT, interleukin-6 (IL-6), HBP, C-reactive protein (CRP), serum resistin, and other indicators have been used clinically to assess the severity and prognosis of sepsis [4,5,6,7,8]. Unfortunately, many primary hospitals in developing countries have not monitored these indicators. In addition, these indicators often require the repeated collection of venous blood for monitoring, which not only increases the pain of neonates but also the financial burden of the family. At the same time, sepsis is often accompanied by anemia, and the dynamic monitoring of the above indicators may further aggravate the severity of anemia, and repeated blood draws increase the incidence of nosocomial infection. As a basic test project carried out in all levels of hospitals in developing countries, blood cell analysis has the characteristics of low blood requirement, inexpensive and little damage to neonates. Our results support the use of these low-cost biomarkers in the assessment of the prognosis of patients with sepsis.

MPV describes the average size of platelets in a blood sample, which is a simple, economical, and useful diagnostic marker for children and neonatal sepsis [16,17,18,19]. Many studies have shown that MPV can predict mortality in adults and children [20,21,22,23]. However, a few studies have focused on the relationship between MPV and the mortality of neonatal, especially premature neonates. Go et al. [24] found that MPV ≥10.2 fL correlates with mortality among neonates born after <32 weeks’ gestation. Hebatallah [25] found that day 3 MPV can be used as a surrogate marker for the prediction of early-onset sepsis and associated mortality in preterm neonates. Regrettably, as of now, there are no studies on MPV and mortality of premature neonates with late-onset sepsis. Our study found that the MPV in the non-survivor group was higher than that of the survivor group. At the same time, logistic regression analysis revealed that MPV was an independent predictor for the prognosis of sepsis. So, we speculate that MPV could represent a relevant predictive marker of mortality in premature neonates with late-onset sepsis. While precise pathophysiologic mechanisms remain elusive, platelet average size may increase in conjunction with inflammation [26]. Moreover, elevated MPV may be indicative of oxidative stress in newborns [27]. In other words, elevated MPV in preterm newborns can inform clinicians of possible hypercoagulative states, increased inflammatory response, and oxidative stress. Among these, the most possible explanation for the relationship between MPV and mortality is an inflammatory response.

RDW is a measure of erythrocyte size variability and has been shown a robust predictor of the risk of all-cause patient mortality and bloodstream infection (BSI) and may reflect overall inflammation, oxidative stress, or arterial underfilling in the critically ill [28]. RDW has been associated with the severity and/or mortality in many diseases of adults, including sepsis and septic shock [13,14,28,29]. Hu et al. [30] speculated that RDW could be used as a prognostic index in septic patients. However, there are a few studies on RDW and the prognosis of premature neonates with sepsis. Our study found that the RDW level of the non-survivor group was significantly higher than that of the survivor group, suggesting that high RDW was a risk factor for sepsis death. Although the underlying mechanism is still unclear, a possible pathophysiologic explanation is that RDW is a surrogate of inflammation, which is known to increase RDW. Several studies found RDW to be associated with blood markers of inflammation, such as interleukin-6, CRP, as well as impaired iron mobilization [31,32]. Also, oxidative stress has been shown to increase anisocytosis by disrupting erythropoiesis and to alter blood cell membrane deformability and red blood cell circulation half-life, ultimately leading to increased RDW [31,33,34].

Further logistic regression showed that MPV and RDW were independent predictors of prognosis, and high levels of MPV and RDW were independent risk factors for death in children with sepsis. Chan [10] revealed that an increase in MPV during the first 72 h of hospitalization was an independent risk factor for adverse clinical outcomes. Takatoshi [11] found that MPV elevation after BSI was identified to be a negative prognostic factor for BSI. Furthermore, Wang et al. [35] showed that RDW was an independent risk predictor of in-hospital mortality in elderly patients with sepsis. As such, we speculate that elevated MPV and RDW levels are independent risk factors for death in children with sepsis.

By comparing the ROC curves of MPV and RDW, the results showed that the AUC was 0.854 for MPV combined with RDW, which suggests that MPV (cut-off value: 11.70) combined with RDW (cut-off value: 15.80) was better for predicting the prognosis of sepsis. A careful analysis of the MPV and RDW in the NICU, which is easily accessible, fast, and affordable, represents a valuable tool to highlight the prognosis of sepsis and thus to quickly start appropriate therapy.

However, this study has some limitations. The first was its retrospective design, suggesting that there may have been unmeasured potential confounders. Meanwhile, the sample size of LOS neonates (only 10 non-survivors) was small, limiting definitive conclusions. Finally, we only report on the situation in China. Our results may not correlate with those of other countries on the grounds of racial differences, as well as different healthcare systems and medical technologies.

## 5 Conclusion

MPV and RDW were independent predictors of prognosis and the combination of the two helps in predicting the prognosis of premature neonates with late-onset sepsis in the early stage.
